# The Metropolitan Hospital Sunday Fund

**Published:** 1912-08-03

**Authors:** 


### THE METROPOLITAN HOSPITAL SUNDAY FUND.

### Report and Awards of the Distribution Committee.

A meeting of the Council of this Fund was held at the Mansion House on Tuesday, July 30, under the presidency of the Right Hon. the Lord Mayor (Sir Thomas Boor Crosby, M.D.).

### Report of the Committee of Distribution.

Sir William Church, in moving the adoption of this Report, said it would be within the recollection of the Council that there were certain questions asked at the general meeting last year with regard to the grounds on which the Distribution Committee had made the grant to St. George's Hospital. There was a good deal of discussion at that meeting, and the Lord Mayor very kindly, and in the interests of the Fund, took upon himself to give an undertaking to the constituents of the Fund that he would examine into the question whether it was correct that some of the money granted to that hospital was devoted to the medical school. At great personal labour and inconvenience the Lord Mayor paid a visit to St. George's Hospital and made a report thereon. In that report one passage aptly summarised the matter: "I am of opinion, after an investigation of all the facts, that the proportionate amount paid by the hospital and school for the upkeep of the laboratories is a fair and proper one, and that the funds expended upon these departments by the hospital are properly taken from the general funds, being for the direct treatment of the patients." The Distribution Committee felt that they were fully empowered to act upon that judgment which the Lord Mayor arrived at, and so there was no hesitation in their minds about allocating a grant to St. George's Hospital this year. It was usual to take the Report as read, but if it were desired he would read it.

Sir John Bell seconded, and expressed the Council's deep gratitude to Mr. William Herring for continuing the magnificent donation of £1,000 a year since his brother's death. He also expressed deep regret at the decease of Mr. Hale, which was a loss to the institution.

The Report was carried unanimously.

Sir Ernest Tritton, Bart., proposed that the Secretary be authorised to purchase convalescent letters up to the value of £100. The reason for that resolution, he explained, was that the Secretary had informed him that owing to the great pressure experienced he had been compelled to obtain letters in advance during the last nine months—in other words, to borrow them against the grants of this Committee to-day. It was an exceptional course, and he hoped it might not be necessary again.

Sir A. Pearce Gould, K.C.V.O., seconded the motion, not from the financial but from the surgical side. The hospital with which he was connected had, happily, like some others, a convalescent home of its own, and he had had daily experience of the value of such a home connected with a hospital. Something was done for patients in hospital, but Convalescent Homes did much to consolidate and increase the benefits obtained from hospital treatment.

The resolution was carried unanimously.

### Inquiry into the Cost per Out-patient.

Sir William Church proposed the following resolution: "The Committee of Distribution, having been informed that the sum allowed by them as the average cost per out-patient attendance has given rise to some dissatisfaction, recommend that the question be again investigated before next year's distribution. The Council therefore requests the Distribution Committee to make such investigation, and for this purpose empower them: (1) to add to their number any gentlemen whose advice and assistance may be desirable; (2) to incur any reasonable expense in the examination of the accounts of the out-patient departments of the hospitals." He said the Distribution Committee were aware that there was a certain amount of dissatisfaction among the Metropolitan hospitals, as they found that the average cost of their out-patients exceeded the amount which the Fund had for many years settled upon. He believed the grounds for such dissatisfaction were sound. From his own knowledge he did not doubt that the cost of out-patient treatment at hospitals had largely increased of late years. In all the large hospitals of London there had grown up special departments which did not exist at the time that the Distribution Committee of the Fund determined the tariff for out-patients. He did not think the rate had been increased since the early years of the Fund, although about eight years ago a committee reported to the Council on the whole subject of out-patients. The cost, particularly in the case of those attending special departments, had very largely increased. The means which had to be used for the treatment of diseases in special departments in most cases involved a large expenditure, instrumental and other, and needed a considerable number of nurses in attendance, which again increased the cost. He considered the question a very important one. It would be desirable to see if the accounts showing cost of out-patients could be classified in some way so as to arrive at a working average amount. To enable the Committee to do that, he proposed that they be empowered to incur any reasonable expense. It would be impossible for the Secretary and his officers to do that extra work; it would be admitted that the Fund was not over-staffed. And it would be well for the accounts in question to be examined by a quite independent outside auditor. By seeing what was about the average cost he hoped the Council would be able to devise means which would allay the dissatisfaction that he knew to exist among managers of Metropolitan hospitals on the point.

Sir Edward Durning Lawrence seconded the motion with great pleasure. He said he had devoted a considerable part of his time to visiting various hospitals, and he knew a good deal about the increasing cost—the almost daily increasing cost—in attending to out-patients in the way they needed. Such an inquiry was an urgent necessity, and he hoped it would be carried.

The Lord Mayor said the words of the seconder "in the way they needed" were of significant import. If hospitals were to be conducted they must be conducted on modern principles. Since he had charge of some portion of St. Thomas's Hospital, nearly sixty years ago, things had entirely changed; it was not now a case of a drug shop and the scalpel, but a lot of incidental and other addenda of expensive character were necessary. Therefore he would support the resolution as far as he could. The out-patient department was a very important one, and though it was also the one most abused, he did not think this abuse would ever be quite got to the bottom of. It was too large a subject to investigate the claims of every person who came for out-patient attention, and it was better to err on the right side in doing all they could in the best manner possible to the credit of the Fund and of the particular institution.

The resolution was carried unanimously.

THE DISTRIBUTION COMMITTEE'S WORK.

Mr. Andrew Motion proposed : " That the thanks of the Council be and are hereby accorded to the Committee of Distribution for the care they have bestowed in the preparation of the awards." He paid a high tribute to the work of the Committee, the names of some of whom were household words.

Mr. F. C. Carr-Gomm seconded, remarking that it was the Distribution Committee who really did the work, and the glory of it was suffused over the whole Council.

The resolution was carried unanimously.

The Hon. Sydney Holland proposed : " That the thanks of the Council be and are hereby given to the editors of newspapers who have pleaded the needs of the hospitals and advocated the cause of the Fund." He wished it to be understood that the resolution was no mere form of words, but expressed real thanks. The Fund had never failed to receive very active and loyal help from every single newspaper.

Mr. J. H. Buxton seconded, remarking that there was evidence that the public was now taking more interest in hospitals than formerly.

This was carried unanimously.

Prebendary Perry proposed : " That the cordial thanks of the Council be and are hereby given to the Right Hon. Sir Thomas Boor Crosby, M.D., F.R.C.S., Lord Mayor, who, as President and Treasurer, has devoted his valuable time and influence to the well-being of the Fund." He spoke of the Lord Mayor's activity in hospital concerns, some of which was within his own sphere of observation.

The resolution was carried without dissent.

The Lord Mayor, in acknowledging the vote, confessed that the work was to him a labour of love. He was always pleased to lend what aid he could to those engaged in his profession—the members of which did so much for so little reward in furtherance of the great work of providing for the poor the advice which they could get nowhere else because they could not afford to pay for it.

### REPORT OF THE COMMITTEE OF DISTRIBUTION TO THE COUNCIL.

The Committee of Distribution held their first meeting on Tuesday, May 16, and then and at subsequent meetings examined the reports and income and expenditure accounts of the several institutions seeking to participate, and made their awards in conformity with Law V.

The Committee beg to report that the total of the Fund to August 2 amounts to £52,300, which, added to the £5,661 distributed last December, brings the total for the year up to £57,961.

The Fund has received a legacy of £20 from the estate of the late Dr. Hermann Adler, Chief Rabbi.

This year 257 institutions have applied for grants from the Fund, being two less than in 1911, and the Committee now recommend the distribution of £52,294 to the several hospitals, institutions, dispensaries, and nursing associations as shown in the appendix hereto.

The Committee having reduced the bases of award to 21 institutions, the governing bodies of the same were informed thereof in accordance with Law V. Thirteen deputations attended the Committee, and, after hearing their several explanations, the awards were finally determined.

The Committee have been informed that it is the intention of the Brixton Dispensary to participate in the proceeds of Sunday kinematograph shows, but to the best of their information nothing has yet been received from this source by the dispensary. The Committee, while recommending an award this year, are of opinion that the governing body of the dispensary should be informed that no grant can be made in future if their intention is carried into effect.

Seven and a-half per cent. of the total sum available for distribution is appropriated to the purchase of surgical appliances during the ensuing year, and $2\frac{1}{2}$ per cent. for district nursing associations.

In compliance with an order of the Council, statistics have been prepared as usual for the special use of its members, showing the number of beds in hospitals, the cost of patients at hospitals and dispensaries, the proportionate expense of management to maintenance, and other valuable information. A copy thereof has been sent to each member of the Council.

THOS. BOOR CROSBY, Lord Mayor, President and Treasurer.

| | |
|---|---|
| DAVID ANDERSON. | JOSEPH C. DIMSDALE. |
| JOHN C. BELL. | ROBERT GREY. |
| F. A. BEVAN. | G. H. MAKINS. |
| WILLIAM S. CHURCH. | F. H. NORMAN. |
| SAVILE CROSSLEY. | E. PARKER YOUNG. |

Mansion House, E.C., July 19, 1912.

### Awards Recommended by the Committee of Distribution for the Year 1912.

THIRTY-THREE GENERAL HOSPITALS.

Charing Cross Hospital, £941 2s. ; French Hospital, £326 1s. 7d. ; German Hospital, £504 3s. 4d. ; Great Northern Central Hospital, £1,186 14s. 4d. ; Guy's Hospital, £1,572 2s. 10d. ; Hampstead and North-West London Hospital, £687 4s. 6d. ; Italian Hospital, £214 6s. 7d. ; Kensington General Hospital, £89 15s. 10d. ; King's College Hospital, £1,372 19s. 5d. ; London Hospital, £5,693 15s. 5d. ; London Homœopathic Hospital, £369 13s. 10d. ; Phillips' Memorial Homœopathic Hospital, £46 2s. 2d. ; London Temperance Hospital, £630 0s. 1d. ; Metropolitan Hospital, £997 14s. 8d. ; Mildmay Hospital, £207 6s. 3d. ; Mildmay Memorial Hospital, £41 18s. ; Miller Hospital and Royal Kent Dispensary, £299 8s. 6d. ; Poplar Hospital, £555 16s. 11d. ; Prince of Wales' General Hospital, Tottenham, £788 18s. 6d. ; Royal Free Hospital, £1,252 4s. 7d. ; St. George's Hospital, £1,581 9s. 9d. ; SS. John and Elizabeth Hospital, £114 11s. 5d. ; St. John's Hospital, Lewisham, £189 3s. 9d. ; St. Mary's Hospital, £1,921 4s. 8d. ; St. Thomas's Hospital, £102 4s. 8d. ; Seamen's Hospital Society, £1,562 0s. 3d. ; The Middlesex Hospital and Convalescent Home, £2,107 13s. 11d. ; University College Hospital, £1,896 18s. 10d. ; Walthamstow, etc., Hospital, £121 11s. 11d. ; Wandsworth, Bolingbroke Hospital, £337 8s. 9d. ; West Ham Hospital, £549 5s. 9d. ; West London Hospital, £1,134 4s. 7d. ; Westminster Hospital, £995 0s. 5d.

SPECIAL HOSPITALS.

SIX CHEST HOSPITALS.—City of London Hospital for Diseases of the Chest, Victoria Park, £1,015 8s. 5d. ; Hospital for Consumption, Brompton, £2,536 4s. 7d. ; Mount Vernon Consumption Hospital, Hampstead, £1,279 4s. 5d. ; Royal Hospital for Diseases of the Chest, £501 10s. 8d. ; Royal National Sanatorium, £70 1s. 4d. ; Royal National Hospital for Consumption, £213 14s. 4d.

Twenty Children's Hospitals.—Alexandra Hospital for Hip Disease, £294 0s. 4d.; Banstead Surgical Home, £21 15s. 10d.; Barnet Home Hospital, £37 12s. 3d.; Belgrave Hospital for Children, £160 9s. 6d.; Cheyne Hospital for Incurable Children, £111 18s. 7d.; East London Hospital for Children, £787 19s. 1d.; Evelina Hospital for Sick Children, £172 4s. 6d.; Home for Incurable Children, £43 3s. 6d.; Home for Sick Children, Sydenham, £108 9s. 8d.; Hospital for Sick Children, £1,154 1s. 2d.; Infants' Hospital, Westminster, £192 13s. 10d.; Kensington, for Children, and Dispensary, £83 1s. 9d.; Paddington Green Hospital for Children, £218 8s. 9d.; Queen's Hospital for Children, £882 3s. 10d.; Royal Waterloo Hospital for Children and Women, £420 4s. 10d.; St. Mary's Hospital, Plaistow, £223 14s. 5d.; St. Monica's Hospital, Brondesbury, £66 6s. 7d.; Victoria Hospital for Children, Chelsea, £647 19s. 5d.; Victoria Home, Margate, £30 8s. 9d.; Hospital for Hip Disease, Sevenoaks, £55 11s. 4d.

Eight Lying-in Hospitals.—British Lying-in Hospital, Endell Street, £121 11s. 2d.; City of London Lying-in Hospital, £180 3s.; Clapham Maternity Hospital and Dispensary, £38 4s. 4d.; East End Mothers' Home, £67 7s. 4d.; General Lying-in Hospital, Lambeth, £179 11s.; Plaistow Maternity Hospital, £49 11s. 11d.; Queen Charlotte's Lying-in Hospital, £507 4s. 10d.; Home for Mothers and Babies, Woolwich, £47 2s.

Five Hospitals for Women.—Chelsea Hospital for Women, £292 2s. 6d.; Hospital for Women, Soho Square, £267 19s.; Grosvenor Hospital for Women and Children, £129 13s. 5d.; New Hospital for Women, £276 5s. 9d.; Samaritan Free Hospital, £361 7s. 1d.

### TWENTY-FIVE OTHER SPECIAL HOSPITALS.

Cancer Hospital, Brompton, nil; Middlesex Hospital, Cancer Wing, £192 8s. 6d.; London Fever Hospital, Islington, £256 9s. 2d.; Gordon Hospital for Fistula, £31 12s. 7d.; St. Mark's Hospital for Fistula, £298 9s. 5d.; National Hospital for Diseases of the Heart, £106 19s. 5d.; Female Lock Hospital, Harrow Road, £263 5s. 8d.; Hospital for Epilepsy, Paralysis, and other diseases of the Nervous System, £175 13s. 4d.; National Hospital for the Paralysed and Epileptic, £823 4s. 3d.; West End Hospital for Diseases of the Nervous System, £420 9s. 6d.; Central London Ophthalmic Hospital, £133 13s. 10d.; Royal Eye Hospital, £273 5s. 2d.; Royal London Ophthalmic Hospital, £843 7s. 8d.; Royal Westminster Ophthalmic Hospital, £62 4s. 8d.; Western Ophthalmic Hospital, £92 1s. 2d.; Royal National Orthopædic Hospital, £426 8s. 5d.; Royal Sea Bathing Hospital, Margate, £141 1s.; St. John's Hospital for Diseases of the Skin, £6 6s. (award December, 1911); St. Peter's Hospital for Stone, £133 12s.; Central London Throat and Ear Hospital, £20 10s. 10d.; London Throat Hospital, £30 7s. 1d.; Metropolitan Ear, Nose, and Throat Hospital, £19 5s. 5d.; Royal Ear Hospital, Dean Street, £51 9s. 9d.; Royal Dental Hospital of London, £250 10s.; National Dental Hospital, £49 1s. 6d.

### THIRTY-FOUR CONVALESCENT HOSPITALS.

Metropolitan Convalescent Institution, Walton, £325 10s. 4d.; Metropolitan Convalescent Institution, Broadstairs, £215 13s. 6d.; Metropolitan Convalescent Institution, Bexhill, £410 12s.; All Saints' Convalescent Hospital, Eastbourne, £341 18s. 10d.; All Saints' Convalescent Home, St. Leonards, £21 7s. 5d.; Ascot Priory Convalescent Home, £72 13s. 3d.; Brentwood Convalescent Home for Children, £11 16s.; Charing Cross Hospital Convalescent Home, £61 5s. 11d.; Chelsea Hospital for Women Convalescent Home, St. Leonards, £51 4s. 2d.; Deptford Medical Mission Convalescent Home, £16 8s. 2d.; Mrs. Gladstone's Convalescent Home, Mitcham, £68 4s. 5d.; Friendly Societies' Convalescent Home, Dover, £85 9s. 9d.; Hahnemann Convalescent Home, Bournemouth, £25 12s. 11d.; Hanwell Convalescent Home, £7 18s. 11d.; Hastings, Fairlight Convalescent Home, £41 16s. 4d.; Hendon, Ossulston Home, £42 12s. 11d.; Herbert Convalescent Home, Bournemouth, £14 15s. 8d.; Herne Bay Baldwin-Brown Convalescent Home, £51 5s. 10d.; Homœopathic Hospital Convalescent Home, £5 5s. 11d.; Hemel Hempstead Convalescent Home, £23 13s. 8d.; Mrs. Kitto's Convalescent Home, Reigate, £42 14s. 10d.; London Hospital Convalescent Home, Tankerton, £59 16s. 10d.; Mary Wardell Convalescent Home for Scarlet Fever, £47 8s. 9d.; Police Seaside Home, Brighton, £43 6s. 7d.; St. Andrew's Convalescent Home, Clewer, £77 15s. 6d.; St. Andrew's Convalescent Home, Folkestone, £138 9s. 2d.; St. John's Home for Convalescent and Crippled Children, £30 0s. 1d.; St. Joseph's Convalescent Home, Bournemouth, £28 3s. 11d.; St. Leonards-on-Sea Convalescent Home for Poor Children, £81 12s. 7d.; Eversfield, St. Leonard's, £29 10s.; St. Mary's Convalescent Home, Broadstairs, £128 4s. 7d.; St. Mary's Convalescent Home, Shortlands, £15 9s. 6d.; St. Michael's Convalescent Home, Westgate-on-Sea, £28 7s. 7d.; Seaside Convalescent Hospital, Seaford, £73 1s. 8d.

### TWENTY-FOUR COTTAGE HOSPITALS.

Acton Cottage Hospital, £111 18s. 3d.; Beckenham Cottage Hospital, £76 1s. 11d.; Blackheath and Charlton Cottage Hospital, £76 15s. 5d.; Bromley, Kent, Cottage Hospital, £98 16s. 2d.; Bushey Heath Cottage Hospital, £29 6s. 4d.; Canning Town Cottage Hospital, £74 15s. 10d.; Chislehurst, Sidcup, and Cray Cottage Hospital, £57 9s. 3d.; Coldash Cottage Hospital, £25 4s. 6d.; Ealing Cottage Hospital, £83 15s. 10d.; East Ham Cottage Hospital, £58 18s. 6d.; Eltham Cottage Hospital, £59 9s. 9d.; Enfield Cottage Hospital, £71 7s. 5d.; Epsom and Ewell Cottage Hospital, £37 12s. 3d.; Hounslow Cottage Hospital, £35 3s. 5d.; Livingstone Dartford Cottage Hospital, £84 12s. 11d.; Kingston, Victoria Hospital, £40 4s. 6d.; Reigate and Redhill Hospital, £121 9s. 6d.; Sidcup Cottage Hospital, £33 15s. 6d.; Teddington, £70 19s. 2d.; Tilbury (Passmore Edwards) Cottage Hospital, £35 0s. 8d.; Willesden Cottage Hospital, £91 14s. 5d.; Wimbledon Cottage Hospital, £48 17s. 7d.; Wimbledon (South) Cottage Hospital, £41 19s. 5d.; Wood Green Cottage Hospital, £65 11s. 5d.

### THIRTEEN INSTITUTIONS.

Hospital for Invalid Gentlewomen, £86 17s. 6d.; St. Saviour's Hospital and Nursing Home, £80 8s. 10d.; Invalid Asylum, Stoke Newington, £29 15s.; Firs Home, Bournemouth, £32 9s. 8d.; St. Catherine's Home, Ventnor, £15 11s. 1d.; Friedenheim Hospital for the Dying, £203 8s. 10d.; Free Home for Dying, Clapham, £143 19s. 11d.; St. Luke's House, Pembridge Square, £137 10s. 11d.; Santa Claus Home, Highgate, £56 12s. 1d.; Royal Mineral Water Hospital, Bath, £51 5s. 10d.; Winifred House, Holloway, £53 1s. 8d.; All Saints, Highgate, £32 3s.; Erskine House, Hampstead, £28 2s. 6d.

### FIFTY-NINE DISPENSARIES.

Battersea Provident Dispensary, £187 4s. 7d.; Billingsgate Dispensary, £44 0s. 7d.; Blackfriars Provident Dispensary, £16 4s. 10d.; Bloomsbury Provident Dispensary, £13 13s. 7d.; Brixton, etc., Dispensary, £41 16s. 1d.; Brompton Provident Dispensary, £17 1s. 11d.; Buxton

Street Dispensary, £12 18s. 2d.; Camberwell Provident Dispensary, £64 19s. 5d.; Camden Town Provident Dispensary, £12 16s. 5d.; Chelsea, Brompton and Belgrave Dispensary, £3 5s. 6d. (award December, 1911); Chelsea Provident Dispensary, £11 19s. 4d.; Child's Hill Provident Dispensary, £9 9s. 9d.; City Dispensary, £59 3s. 1d.; Clapham General and Provident Dispensary, £21 7s. 5d.; Deptford Medical Mission, £21 7s. 5d.; Eastern Dispensary, £32 18s. 1d.; East Dulwich Provident Dispensary, £54 9s. 2d.; Farringdon General Dispensary, £38 11s. 1d.; Finsbury Dispensary, £40 13s. 11d.; Forest Hill Provident Dispensary, £32 8s.; Greenwich Provident Dispensary, £24 17s. 6d.; Hackney Provident Dispensary, £20 8s. 7d.; Hampstead Provident Dispensary, £46 4s. 11d.; Holloway and North Islington Dispensary, £11 12s. 7d.; Islington Dispensary, £43 13s. 7d.; Islington Medical Mission, £40 13s. 11d.; Kennington and Vauxhall Provident Dispensary, £11 19s. 4d.; Kensal Town Provident Dispensary, £11 0s. 7d.; Kentish Town Medical Mission, £19 13s. 2d.; Kilburn, Maida Vale and St. John's Wood Dispensary, £30 0s. 1d.; Kilburn Provident Medical Institution, £43 10s. 3d.; London Dispensary, Spitalfields, £11 8s. 11d.; London Medical Mission, Endell Street, £82 8s.; Margaret Street, for Consumption, £34 9s. 3d.; Metropolitan Dispensary, £45 16s. 3d.; Mildmay Medical Mission Dispensary, £9 8s. 1d.; Notting Hill Provident Dispensary, £17 15s. 8d.; Paddington Provident Dispensary, £30 15s. 6d.; Public Dispensary, Drury Lane, W.C., £32 9s. 8d.; Queen Adelaide's Dispensary, £22 4s. 6d.; Royal General Dispensary, £25 14s. 7d.; Royal Pimlico Provident Dispensary, £31 15s. 11d.; Royal South London Dispensary, £40 17s. 4d.; St. George's Hanover Square, Dispensary, £28 19s. 7d.; St. John's Wood Provident Dispensary, £39 6s. 6d.; St. Marylebone General Dispensary, £45 13s. 2d.; St. Pancras and Northern Dispensary, £32 11s. 8d.; South Lambeth, Stockwell, and North Brixton Dispensary, £21 3s. 9d.; Stamford Hill, Stoke Newington Dispensary, £46 3s. 3d.; Tower Hamlets Dispensary, £33 10s. 2d.; Walworth Provident Dispensary, £12 16s. 5d.; Wandsworth Common Provident Dispensary, £10 5s. 2d.; Westbourne Provident Dispensary, £15 6s. 1d.; Western Dispensary, £68 7s. 9d.; Western General Dispensary, £77 3s. 9d.; West Ham Provident Dispensary, £10 18s. 11d.; Westminster General Dispensary, £40 17s. 3d.; Whitechapel Provident Dispensary, £29 3s.; Woolwich Provident Dispensary, £34 5s. 7d.

THIRTY-TWO NURSING ASSOCIATIONS.

Belvedere, Abbey Wood, £6; Brixton, £18; Central St. Pancras, £18; Chelsea and Pimlico, £18; Hackney, £24; Hammersmith, £42; Hampstead, £18; Isleworth, £12; Kensington, £42; Kilburn, £6; Kingston, £30; Lambeth Road (Catholic), £12; Metropolitan (Bloomsbury), £12; St. Olave's (Bermondsey), £18; Paddington and Marylebone, £40 16s.; Peckham, £12; Plaistow, £103 16s.; Plaistow (Maternity), £166 16s.; Rotherhithe, £12; Shoreditch, £54; Sick Room Helps Society, £28 16s.; Sidcup, £6; Silvertown, £18; South London (Battersea), £54; Southwark, £52 16s.; South Wimbledon, £36 12s.; Tottenham, £11 8s.; Westminster, £24; Woolwich, £36; East London, £150; North London, £60; London District, £300.

